# Controlling 3D Contractility via Engineered Fibrous Hydrogel Composites

**DOI:** 10.1002/adfm.202524101

**Published:** 2026-02-25

**Authors:** Karen L. Xu, Yuqi Zhang, Alysse DeFoe, Georgios Kotsaris, Brendan Stoeckl, Matthew D. Davidson, Jason A. Burdick, Robert L. Mauck

**Affiliations:** 1 Department of Bioengineering, University of Pennsylvania, Philadelphia, Pennsylvania, USA; 2 Center for Engineering Mechanobiology, University of Pennsylvania, Philadelphia, Pennsylvania, USA; 3 McKay Orthopaedic Research Laboratory, Department of Orthopaedic Surgery, Perelman School of Medicine, University of Pennsylvania, Philadelphia, Pennsylvania, USA; 4 BioFrontiers Institute, University of Colorado Boulder, Boulder, Colorado, USA; 5 Department of Chemical and Biological Engineering, University of Colorado Boulder, Boulder, Colorado, USA

**Keywords:** contraction, electrospun fibers, hyaluronic acid, hydrogels, microtissues

## Abstract

Complex and dynamic mechanobiological crosstalk occurs between cells and their extracellular matrix (ECM) to support contraction, a process required for tissue morphogenesis and wound healing. In vitro models can be used to study this crosstalk by mimicking the ECM (collagen fibers within a ground substance) using controlled environments and defined mechanics. While useful, most in vitro models utilize poorly-defined natural hydrogels that lack independent control over hydrogel properties and contraction tunability. Here, a fully-defined hydrogel composite is introduced consisting of fragmented synthetic fibers (a collagen fiber mimic) that, when embedded within a synthetic hydrogel (a ground substance mimic), supports cell-mediated traction-based contraction in a manner similar to traditional collagen gels. Tuning this composite material by modulating fragmented fiber density and length and embedding hydrogel density and crosslinking enables control over contraction. Cells cultured within contraction-permissive constructs support microtissue cell alignment and local densification of fiber fragments, while culture in contraction-resistant composites (greater embedding hydrogel crosslinking) do not. This innovative composite material expands our ability to interrogate the complex cell-ECM interplay during tissue morphogenesis.

## 1 | Introduction

The morphogenesis of tissues [1], their adult function [2], and their healing after injury [3] all require tissue-level contraction to occur. At the macroscopic level, contraction reflects the summation of endogenous cells either pulling directly on one another or on the nascent or extant extracellular matrix (ECM) that exists between individual cells. As such, ECM properties and extent of remodeling dictate whether cell-generated contraction is reflected in tissue-level changes [4]. For example, contractility is critical for load-bearing tissues in the knee such as the meniscus, a structure dominated by fibrous collagen that resists tensile forces [5]. When myosin-based contractility is eliminated during development, aberrant tissue morphogenesis occurs. To better understand applications of contractility, including the cell-ECM crosstalk that enables fibrous tissue formation in the developing knee, model culture systems that mimic features of the ECM, including environments that exhibit the user-programmed duality of either supporting or resisting contraction are needed.

The ECM in which cells reside is a complex milieu of proteins and polysaccharides [6], which can be modeled as collagen fibers within a ground substance, the amorphous substrate that embeds the fibers together [7]. Unsurprisingly, hydrogels consisting of natural polymers (e.g., collagen, fibrin) [8–10] have been used to model cell-matrix interactions due to their inherent fibrillar and biologic composition, which enables cell-mediated remodeling either by direct (via tension through adhesive residues) or indirect (such as the catabolism of matrix via the action of matrix degrading enzymes) methods. These natural polymers not only assemble into fibrils, but also have native cell-adhesive sites, allowing cells to both attach to and naturally degrade and remodel their environments. When contractile cells are introduced into these fibrillar environments, bulk contraction of microtissues can be visualized with the naked eye. These studies have provided great insights into physiologic processes that rely on contraction, including the identification of cell types and matrix composition that are most conducive to wound healing [11], as well as novel drugs that support healthy cardiac function [12], skeletal muscle contraction [13] or reduce lung fibrosis [14].

While useful in this regard, these natural ECM-based polymers have inherent limitations. For example, such materials are often poorly defined in their composition (as is the case for Matrigel), may suffer from batch-to-batch variations based on their natural origin [15], and are challenging to decouple extracellular inputs (e.g., mechanics and adhesion) given that these features are inextricably linked to one another. For example, collagen gels can be stiffened by simply increasing collagen density, but this inherently changes the biologic environment of resident cells by simultaneously providing more adhesive moieties with which cells can interact. This hinders mechanistic studies to understand how independent features of the ECM may support cell-mediated contraction and other behaviors. To overcome these limitations, we and others have turned toward increasingly sophisticated 3D synthetic systems to study the interplay of cells and their ECM under conditions that support or suppress contraction. Synthetic hydrogels may provide enhanced modularity of the microenvironment, in which cell-adhesive sites, mechanical properties, and matrix density can be directly and independently tuned [16]. However, the covalent crosslinking that is often used to stabilize many of these synthetic systems can hinder cell remodeling [17].

To this end, recent advances in material processing have introduced fibrillar structures found in natural hydrogels such as collagen into synthetic systems to enable the decoupling and therefore independent investigation of extracellular cues. For example, electrospun fibrous dextran methacrylate networks were produced that allowed contraction when cells were seeded atop the fibrous structure [18]. However, control over the contraction was challenging, and the material format did not allow for investigation of cells in 3D. To address this, we recently generated fragmented fibers from electrospun norbornene-modified hyaluronic acid (NorHA) [19]. The added norbornenes enabled orthogonal photocrosslinking, first to form the fiber and attach arginine-glycine-aspartic acid (RGD) cell-adhesive moieties, and second to crosslink the fragmented fibers to one another. This network of weak inter-fiber crosslinks and inherent porosity in the fiber network supported cell remodeling and contraction, which could be tuned through altering the fiber density. This system advanced on current collagen hydrogels by providing a 3D and fully-defined system to support macroscopic cell contraction. However, the ability to provide a contraction-resistant comparison was limited due to the coupling of fiber density with adhesive sites.

**FIGURE 1 |:**
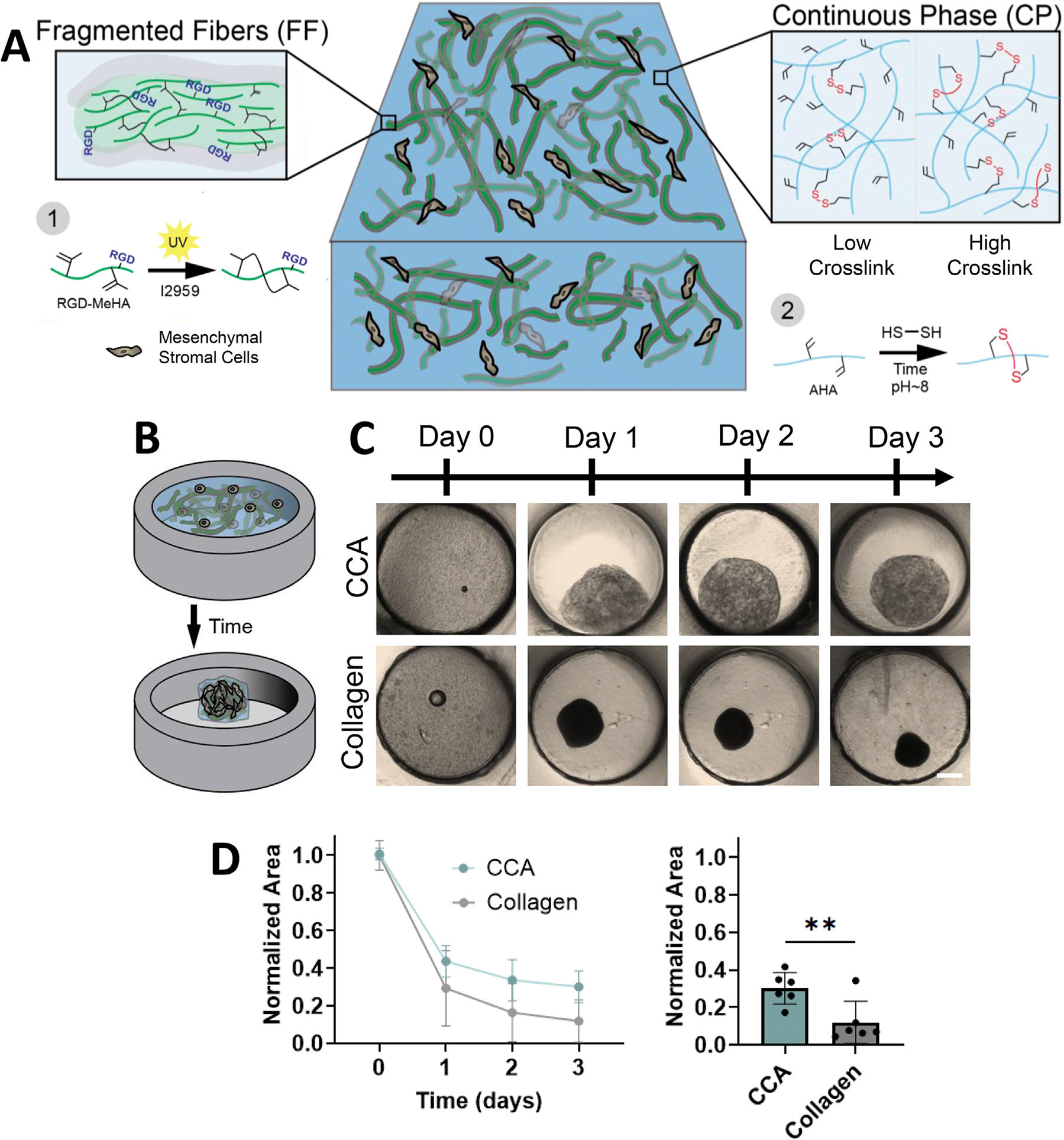
Fabrication and validation of composite contractile assemblies (CCAs). (A) Schematic of CCA fabrication, including the combination of fragmented fibers (FF) made from RGD-modified methacrylated hyaluronic acid (MeHA), continuous phase (CP) made from acrylated hyaluronic acid (AHA), and mesenchymal stromal cells (MSCs), with the full assembly stabilized with dithiolated crosslinkers. Extent of CP-phase acrylate consumption can range from minimal (low crosslink) to extensive consumption (high crosslink). (B–D) Schematic (B), representative images (C, Scale bar = 400 μm), and area quantification (D) of the contraction of a CCA construct (0.7% (w/v) CP; 5% (v/v) FF; 5% acrylate consumption; 5 × 10^6^ cells/mL) versus collagen construct (2.5 mg/mL) over time (D, left panel) and on day 3 (D, right panel). *n*= 5–8 samples per group. **p < 0.01. Two-tailed unpaired Student’s *t*-test on log-transformed data. Data are mean ± s.d.

Here, we sought to overcome this limitation by advancing this framework. We developed a tunable fibrous hydrogel composite which added a weakly crosslinked non-adhesive bulk phase (to mimic the ECM ground substance) to encapsulated fiber fragments modified with adhesive peptides (to mimic collagen fibers) [19], with both components formed from hyaluronic acid (HA) (Figure 1A). In this current study, through the addition of the embedding hydrogel, we tuned the extent to which the scaffold could support or inhibit cell-mediated contraction without altering other biophysical cues such as fiber density or extent of cell adhesion sites. We first demonstrated the tunable nature of the composite, confirming that it displays behaviors characteristic of collagen contraction (e.g., the influence of material density and cell number on contractility), while also highlighting additional functionalities (e.g., the ability to control the extent of contraction). We demonstrated its application as a microtissue scaffold and further explore how cells engage with the fiber fragments within the scaffold and with each other to support contraction. Together, this work introduces an innovative platform that will enable a deeper investigation of the biochemical and biophysical regulators of contraction in biological processes such as tissue morphogenesis or disease progression.

## 2 | Results and Discussion

### 2.1 | Fabrication of Composite Contractile Assemblies (CCAs)

Given that developing tissues and their extracellular matrices are dynamic, our goal was to create a material system in which cell-mediated remodeling could be tuned in terms of both extent and timing but without influencing critical extracellular cues (such as cell-adhesive site concentration or surrounding material density). To accomplish this, we introduced multiple independent constituents (cells and fragmented fibers (FFs)) into a bulk continuous hydrogel whose crosslinking extent could itself be tuned to change CCA properties (Figure 1A). We hypothesized that this design would enable a range of cell remodeling behaviors, including user-defined halting of remodeling.

To establish the CCAs, we included cell-adhesive fragmented fibers (FFs) formed from electrospun methacrylated HA (MeHA) modified with adhesive peptides. We previously showed that cells could contract assemblies of engineered fragmented fibers based on the fiber concentration [19]. These engineered fragmented fibers have advantages over the use of collagen fibers, as the former has a polymer composition that is fully defined, enabling control over individual fiber stiffness and the density of adhesive ligands per unit length of fiber. FF lengths averaged 86.9 μm, which enabled multiple cells in proximity to interact with the same RGD-modified fiber (Figure S1A–C) to support long-range force transmission, similar to that which occurs in fibrillar collagen gels [20]. FF thicknesses averaged 0.97 μm (Figure S1D–F). Our previous work involved sequential photopolymerization of Norbornene-modified HA fragmented fibers to stabilize the fiber and to crosslink the individual fibers to one another to form a network [19]. This resulted in a fiber assembly in which the tunable parameters included fiber density or cell concentration, both of which can also be tuned in collagen hydrogels. However, in the present work, instead of crosslinking the fiber fragments to each other, the FFs were embedded within an acrylated HA (AHA) [13] continuous phase (CP). AHA crosslinking with dithiols provided an independent material phase relative to the embedded FFs, which could be independently tuned. The addition of this CP allows for variation in contraction without varying fiber (and cell-adhesive site) density, unlike in our previous system and in collagen. Given the increased kinetics of the thiol-acrylate reaction compared to the thiol-methacrylate reaction and the assumption that the methacrylates on the MeHA fibers are fully consumed from the initial electrospun fiber UV crosslinking, most of the crosslinking likely occured between the acrylates of the CP as opposed to with the methacrylates on the FF. Ultimately, this allows user-defined covalent crosslinking that does not rely on photopolymerization and control over an additional fiber-embedding continuous phase (which does not include adhesive moieties) to support or resist contraction when cells interact with the co-embedded FFs (through their adhesive RGD ligands).

To confirm contraction capacity, mesenchymal stromal cells (MSCs) were added to the CCAs and the CP was partially crosslinked to 5% consumption of the acrylate groups (Figure 1B). These lightly crosslinked constructs underwent time-dependent contraction on a time-scale similar to collagen-based hydrogels (Figure 1C), with the CCAs exhibiting ~56% area reduction over the first day and ~70% by the third day (Figure 1D). The CCAs did not contract to quite the same extent as collagen over this time period (~70% vs. ~90%, respectively). This may be due to the presence of the CP, which may resist contraction, or due to differences in contractility of cells adhering to RGD vs. collagen-based adhesive moieties. However, compared to previously studied homogenous hydrogels without fibrous components, the CCAs contracted to a greater extent [18]. This is striking, given that the adhesive sites are only found on the FF fraction. Perhaps the cells engage with and pull the FFs either through or with the CP into a contracted construct. Regardless, the lightly-crosslinked CP formed a stable construct but did not interfere with the cell-mediated remodeling supported by the FF fraction. Importantly, removal of the FF population destabilized the construct, suggesting not only its role in allowing cell interactions via RGD-modification but also in stabilizing the CCA. Yet, with slightly increased CP support (0.7% CP to 1% (w/v) CP), the CCA continued to exhibit contraction, which was lost when the FF were removed (Figure S2), suggesting that cells must adhere to the FF to enact macroscopic contraction.

**FIGURE 2 |:**
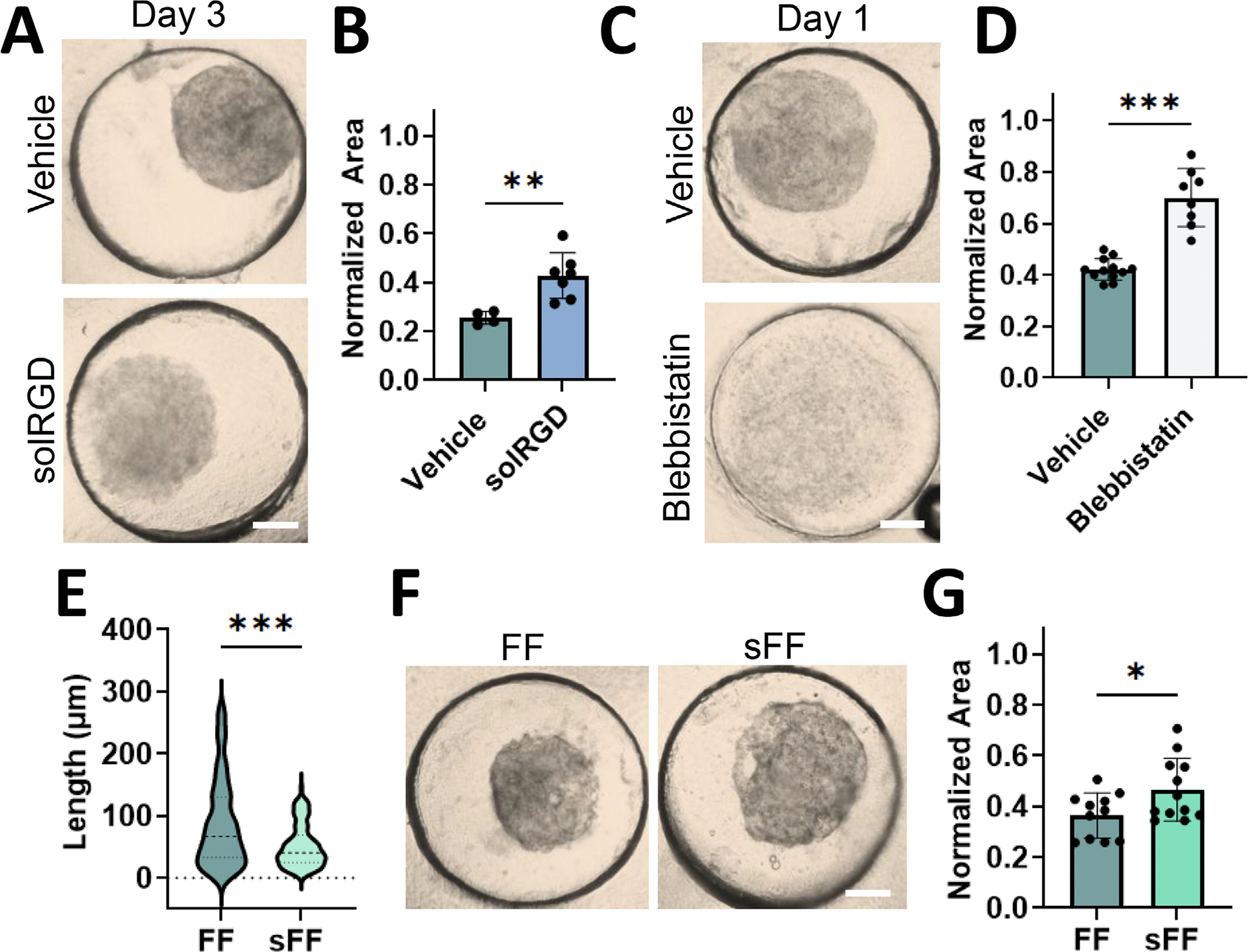
Cells use integrin-based adhesions to adhere to and exert tractions to stabilize surrounding fragmented fibers. (A,B) Representative images (A, Scale bar = 400 μm) and quantification (B) of day 3 vehicle (DMSO) and soluble RGD (solRGD) addition to CCA (0.7% (w/v) CP; 5% (v/v) FF; 5% acrylate consumption; 5 × 10^6^ cells/mL). *n*= 4–7 constructs per group. **p < 0.01. Two-tailed unpaired Student’s *t*-test. (C,D) Representative images (C, Scale bar = 400 μm) and area quantification (D) of CCA treated with vehicle (DMSO) or Blebbistatin. *n*= 5–8 samples per group. ***p < 0.001. Two-tailed unpaired Student’s *t*-test. (E) Quantification of fiber lengths (FF vs. short FF (sFF)). *n*= 138–145 fibers per experimental group. ***p < 0.001. Mann-Whitney test. (F,G) Representative images (F, Scale bar = 400 μm) and quantification (G) of day 3 CCA (0.7% CP; 5% FF or sFF; 5% acrylate consumption; 5 × 10^6^ cells/mL). *n*= 11–12 constructs. *p < 0.05. Two-tailed unpaired Student’s *t*-test. Data are mean ± s.d.

We performed several additional studies to better understand how cells were interacting with and contracting the CCA construct. These studies show that the cells adhere to the FF to stabilize these fibers, given that introducing a competitive inhibitor of integrin binding (soluble RGD) [21] reduced contraction extent (Figure 2A,B). When myosin-mediated contractility was abrogated via blebbistatin, constructs did not contract (Figure 2C,D; Figure S3), highlighting that cells exert traction once adhered to the fibers. Additionally, fragmenting the fibers further so that they were shorter also decreased extent of contraction, likely due to a lower number of cells that could interact with a single fiber (Figure 2E–G). In total, these studies suggest that cells adhere to the fragmented fibers and then exert traction to induce macroscopic contraction through multiple cell-fiber interactions.

Since both the CCAs and collagen support macroscopic contraction, we next explored how these differential extracellular environments influenced cell response at the transcriptional level using RNAseq. Compared to collagen gels, cells in CCAs showed numerous processes that were downregulated (Figure S4A–C), including integrin-mediated signaling pathways, cell-matrix adhesions (ITGB5, ITGA2, ITGA11), and actin-based cell projections (ACTA2). This is unsurprising given the wide range of integrins that have a strong affinity for collagen [22] which can in turn recruit and activate actin cytoskeleton-based contractility mechanisms [23]. Interestingly, ECM organization ontologies showed a mixed response, with downregulation of some genes associated with matrix deposition and turnover (MMP11, COL13A1) and upregulation of others (LOX) associated with matrix crosslinking. This highlights that the ECM is differentially influential in cells seeded in the CCAs relative to collagen. Finally, we noted that cells within CCAs exhibited increased expression of inflammatory markers (TLR2, TLR5, CXCL2), amongst other key biologic processes (Figure S4D,E). This may suggest one of two things: (1) cells in CCAs that have a lower complement of binding moieties may be under stress and so are actively working to remodel their matrix [24] or (2) the low molecular weight hyaluronic acid may itself act as a pro-inflammatory stimulus [25]. Importantly, all trends were consistent across 3 distinct donors (Figure S4C,E). These transcriptomic studies highlight how the ECM influences cell phenotype and the importance of carefully engineering hydrogels to present defined extracellular environments for in vitro studies that are decoupled from adhesion-dominated cellular responses. For example, in pro-inflammatory conditions such as the meniscus following injury, utilizing a hyaluronic-based cell scaffold for in-vitro studies may better mimic in vivo environments.

**FIGURE 3 |:**
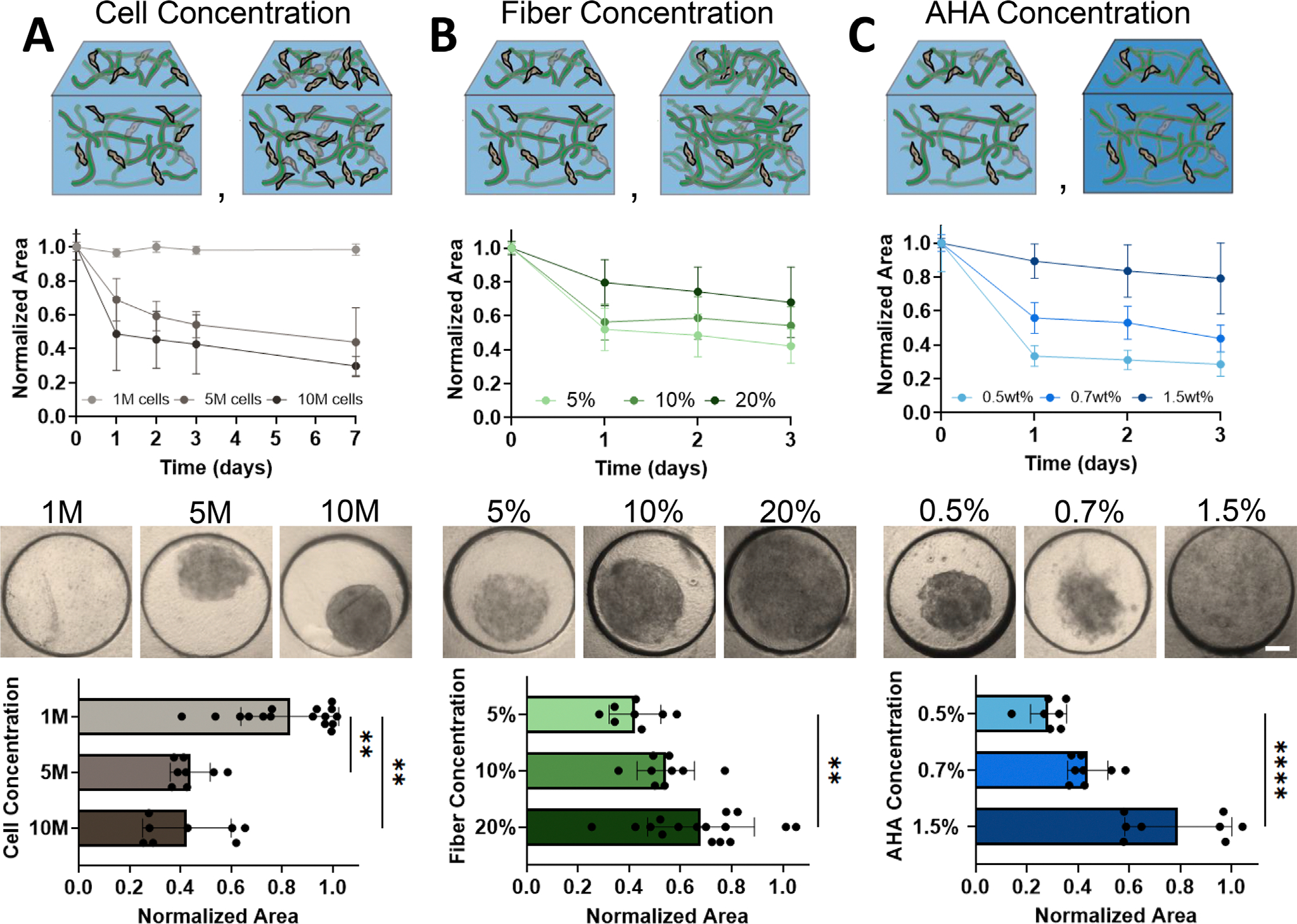
Tuning the contraction of CCAs. (A–C) Schematic depicting parameter change (top panel), quantification of area over time (top middle panel), representative images (bottom middle panel, Scale bar = 400 μm) and quantification at day 3 (bottom panel) of assemblies with varying cell concentration (A, 0.7% (w/v) CP; 5% (v/v) FF; 5% acrylate consumption; 1M (1 × 10^6^), 5M, 10M cells/mL, *n*= 7–16 constructs per group, Kruskall-Wallis with Dunn’s multiple comparisons), FF concentration (B, 0.7% CP; 5, 10, 20% FF; 5% acrylate consumption; 5 × 10^6^ cells/mL, *n*= 8–18 constructs per group, one-way ANOVA with Tukey post-hoc testing), and AHA concentration (C, 0.5, 0.7, 1.5% CP; 5% FF; 5% acrylate consumption; 5 × 10^6^ cells/mL, *n*= 7–8 constructs per group, Kruskall-Wallis with Dunn’s multiple comparisons). **p < 0.01, ****p < 0.0001. Data are mean ± s.d.

Based on this finding, we next explored how tuning different features of CCAs could influence the extent of contractility. To determine how cell concentration regulated the rate of contraction, we first investigated contraction with varying cell densities. Here, we found that a reduction in cell density decreased the rate, but not the final extent, of construct contraction if the initial cell density was above a threshold (Figure 3A). This is unsurprising, given that construct contraction is cell-mediated and that macroscopic contraction is the summation of localized contractions of FF surrounding each cell. These findings are also in line with the cell-density dependent contraction observed in other natural materials (e.g., collagen) [26]. Additional studies may further explore the role of different cell types or states on contraction of the material.

Next, to explore the role of individual fiber fragments, we varied FF concentration from 5% to 20% (v/v) and evaluated composite contraction over 3 days. Results showed that a reduction in fiber concentration resulted in an increased extent of contraction (Figure 3B). These findings are in line with our previous studies suggesting that increasing the FF concentration reduces the potential for cell-mediated densification of the construct (due to the increased density of the surrounding material) [19] and is consistent with previous studies in collagen gels demonstrating that decreased material concentration increases the extent of contraction [26].

We then explored the role of the continuous phase (CP) density on construct contraction, with a focus on identifying the maximal concentration of the CP that continued to support contraction. Concentrations of 0.5% and 0.7% (v/v) supported construct contraction, which confirmed our hypothesis that the CP could stabilize, but not interfere with cellular remodeling. Notably, increasing the CP concentration to 1.5% (v/v) eliminated construct contraction over the first 3 days, likely due to the increase in CP density that dominates over FF cues when composites were formed (Figure 3C). Likewise, at high concentrations, the CP behaved as a bulk synthetic hydrogel, which generally do not enable cell-mediated contraction [19]. Importantly, partial crosslinking was required for construct stabilization, as the construct lost integrity if the dithiol crosslinker was not added (data not shown).

**FIGURE 4 |:**
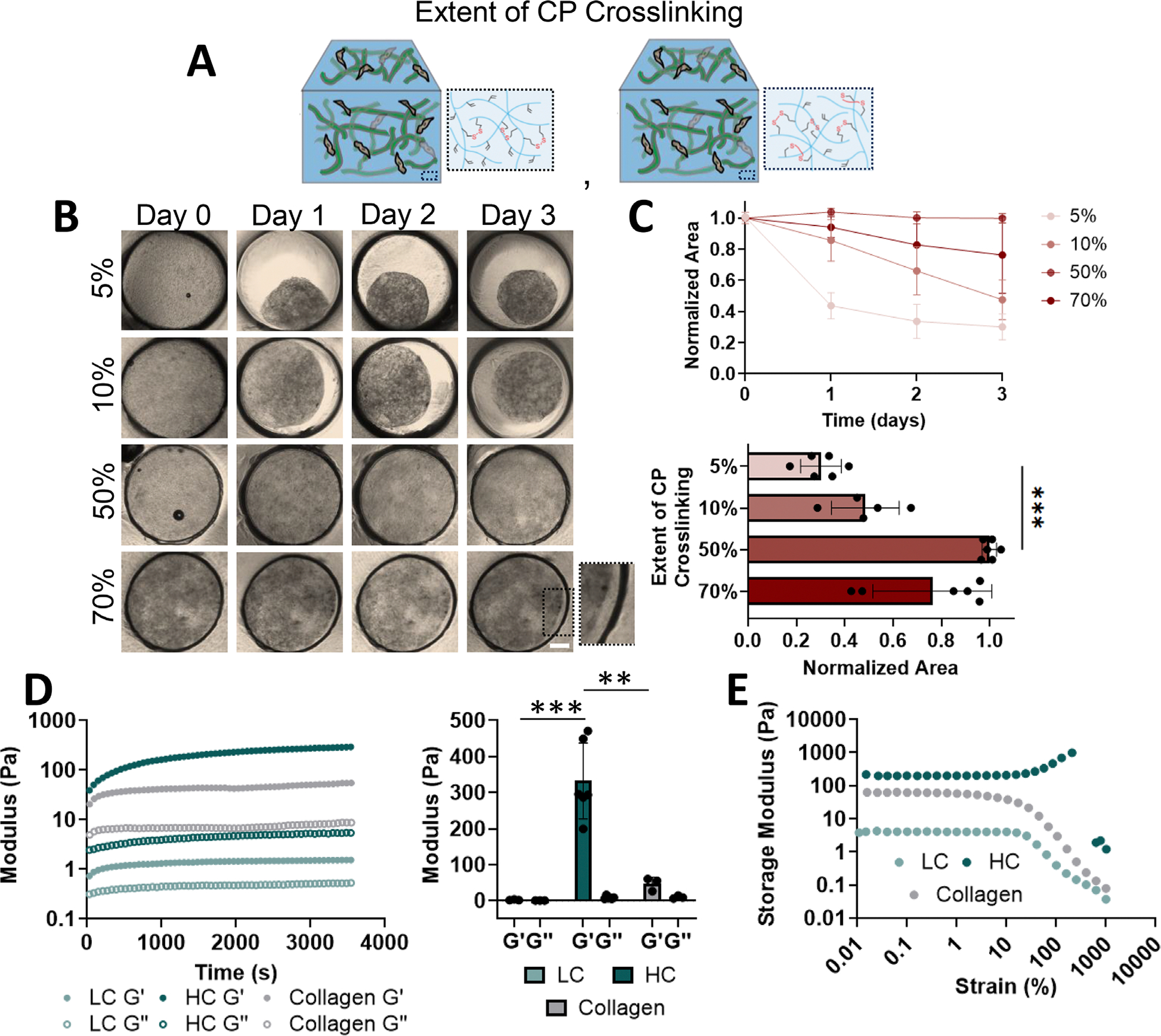
CCA contraction is dependent on CP crosslinking. (A) Schematic depicting tunable CP crosslinking within the CCAs. (B,C) Representative images (B, Scale bar = 400 μm) and quantification over time (C, top panel) and at day 3 (C, bottom panel) of the CCAs with varying CP crosslinking (0.7% (w/v) CP; 5% (v/v) FF; 5%, 10%, 50%, 70% acrylate consumption; 5 × 10^6^ cells/mL). *n*= 5–6 constructs per group. ***p < 0.001. Kruskall-Wallis with Dunn’s multiple comparisons. (D) Representative rheological time sweeps (left panel, storage (G′) and loss (G″) modulus, 1 Hz, 1% strain) and final rheologic mechanical properties of constructs (right panel, LC: 5%, HC: 50% extent of CP crosslinking, which is equivalent to acrylate consumption). *n*= 3–6 constructs per group. **p < 0.01, ***p < 0.001. One-way ANOVA with Tukey post-hoc testing between G’. (E) Representative strain sweeps of constructs (1 Hz, 0.01%–1000% strain). *n*= 3–4 constructs per group. Data are mean ± s.d.

A feature of the CCAs is the ability to stabilize the hydrogel through increasing the crosslinking of the CP phase without altering the cell density. To explore the role of CP crosslinking on contraction, the extent of acrylate crosslinking in the CP was varied by adjusting the concentration of dithiol crosslinker initially added (Figure 4A). Interestingly, the rate and extent of construct contraction depended on the initial crosslinker concentration (Figure 4B,C). With 5% consumption, the most rapid and greatest extent of contraction was observed, likely because the CP was only loosely stabilized, allowing for maximal cell-mediated remodeling. 10% consumption supported similar contraction extents as seen in the 5% group by day 3, but contraction occurred at a slower rate. This could be due to the increased crosslinking that initially slowed remodeling and cell interactions with the embedded adhesive FFs. Since the thioester crosslinks in the CP are susceptible to hydrolytic degradation, degradation of these bonds may reduce extracellular impediments to contraction over time, and ultimately introduce a temporal slowing of contraction that is not possible in natural hydrogels [27]. When the consumption of the CP was set at 50%, no contraction was observed over 3 days. It is not clear whether longer time periods could eventually support remodeling through hydrolytic cleavage of thioester crosslinks. Interestingly, at 70% consumption, we observed minimal, but evident contraction over time. This is perhaps due to a reduction in crosslinking efficiency due to a saturation of exposed acrylate groups by excess thiols, which would result in nonfunctional pendant thiol groups in the material [28]. We observed a near-peak consumption at 50%, perhaps because a portion of the acrylate groups are covered by the FFs or cells, resulting in fewer available acrylate groups than calculated. Altogether, these data support that the contraction extent can be tuned by simply changing the extent of initial CP crosslinking, allowing for a direct comparison of hydrogels that are permissive and resistant to contraction while decoupling cues such as adhesive site concentration and density of the extracellular environment.

To maximize the stability and contraction extent, all subsequent studies were performed with a CP of 0.7% (w/v), FF of 5% (v/v), and cell density of 5 × 10^6^ cells/mL. Crosslinking extent was varied between 5% (or Low Crosslink (LC)) for a macroscopically contractile material versus 50% (or High Crosslink (HC)) for a biochemically-identical (equivalent polymer density, cell-adhesive sites etc.) material that resisted macroscopic contraction. In both groups, the kinetics of the thiol-acrylate reaction enabled hydrogels to fully crosslink within 1 h (Figure 4D) while maintaining cell viability (Figure S5). In the LC group, the CP phase had only minor contributions to the mechanical properties, evidenced by both the storage modulus (G′) and the loss modulus (G″) being within an order of magnitude of each other. Conversely, the increased density of crosslinks in the CP in the HC group resulted in increased stiffness compared to the LC group. Collagen at 2.5 mg/mL exhibited a stiffness between the LC and HC groups. Interestingly, the LC group exhibited negligible strain stiffening, likely because the reduced crosslinking of the CP did not sufficiently couple the fibers to the embedding component (Figure 4E). In contrast, the HC group exhibited a strain stiffening response, potentially due to CP-fiber coupling. This is consistent with the strain-stiffening response seen in our previous study when fragmented fibers were covalently annealed to one another [19]. These varied responses to strain in the CCA groups suggest that linking the embedding phase alters the extent of force propagation through the composite material. Of note, all hydrogel groups remained intact through day 3 (Figure S6), with decreased fluorescence trackers in the LC group over time, potentially due to fewer CP crosslinks compared to the HC group.

**FIGURE 5 |:**
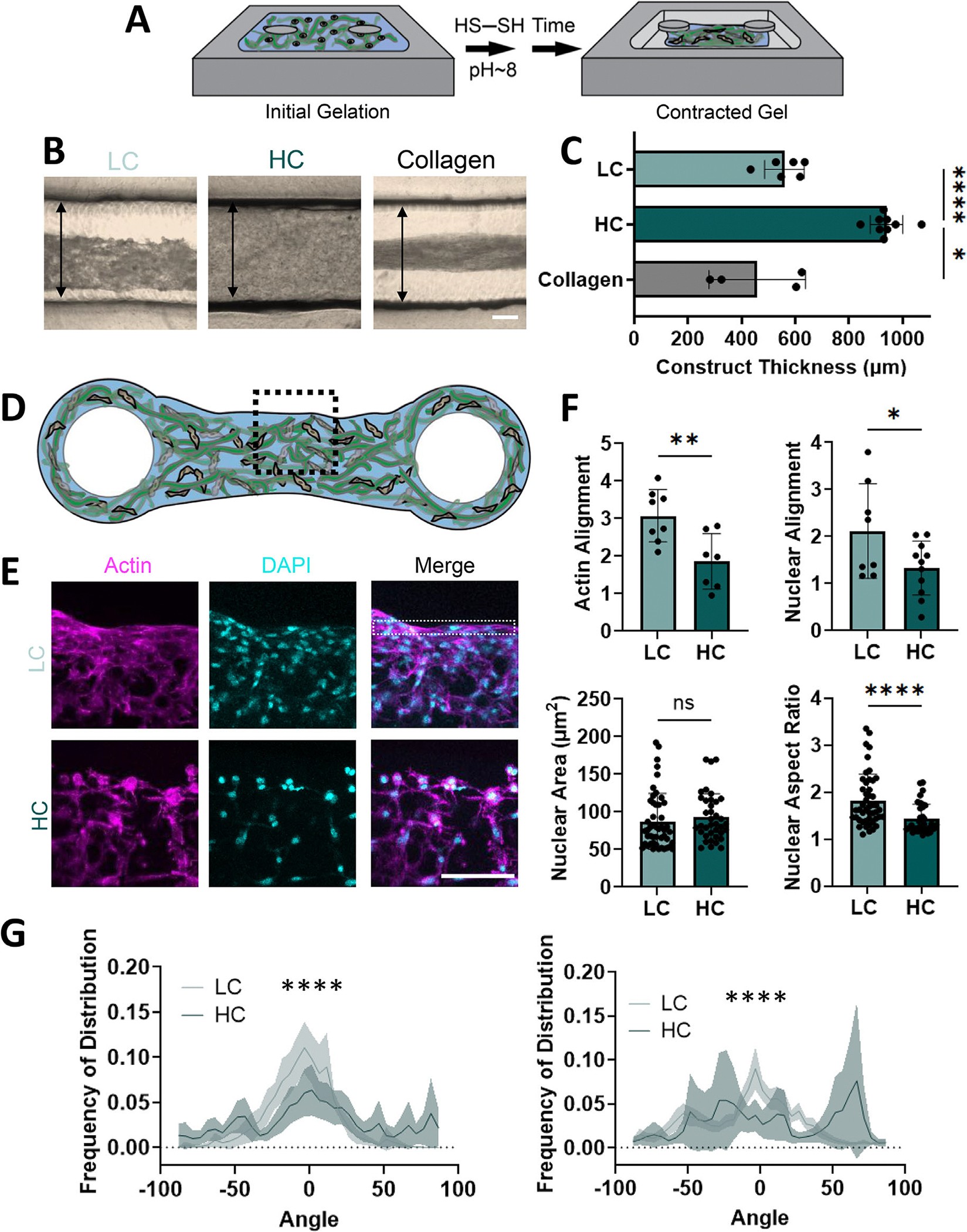
Alignment of CCA microtissues is based on contraction along an axis of tension. (A) Schematic of CCA contraction in PDMS molds between boundary constraints. (B,C) Representative images (B, black arrows represent pre-contraction construct thickness, Scale bar = 200 μm) and quantification (C) of collagen (2.5 mg/mL) and CCAs with varying CP crosslinking (0.7% (w/v) CP; 5% (v/v) FF; LC: 5%, HC: 50% extent of CP crosslinking; 5 × 10^6^ cells/mL) after 3 days. *n* = 4–9 constructs per group. *p < 0.05, ****p < 0.0001. Welch’s ANOVA with Dunnett’s T3 multiple comparison’s test. (D,E) Schematic (D, dashed black line represents location on construct represented in panel E) denoting area of representative max projections (E, dashed white line represents ROIs for analysis in panel F, Scale bar = 100 μm). (F) Quantification of actin alignment (top left panel, *n* = 7–8 constructs per group, Two-tailed unpaired Student’s *t*-test), nuclear alignment (top right panel, *n* = 8–11 constructs per group, Two-tailed unpaired Student’s *t*-test), nuclear area (bottom left panel, *n*= 38–48 cells per group, Mann-Whitney test), and nuclear aspect ratio (bottom right panel, *n* = 35–46 cells per group, Mann-Whitney test). ns indicates no statistical significance, *p < 0.05, **p < 0.01, ****p < 0.0001. (G) Frequency distribution of orientation of actin (left panel) and nuclei (right panel). *n* = 18–23 analyzed ROIs per group. ****p < 0.0001. Watson-Wheeler Test. Shaded lines represent 95% confidence interval. Data are mean ± s.d.

Previous studies have fabricated microtissues as tissue-level mimics of in vivo environments [29] to elucidate the role of contractility [30] and alignment [31] in tissue development and repair [16]. For example, these microtissues have broadly informed cellular remodeling and ECM deposition for tissue healing [32, 33], the influence of pathologic stiffness in hypertrophic cardiomyopathy [34], and cell phenotype responses to localized cellular alignment in a tendon model [35]. While illustrative, these studies were performed in natural ECM-based polymers that are poorly defined and limit independent exploration of how multiple extracellular inputs, such as mechanics and adhesion, influence cell behavior. To show that the CCAs can be used as an alternative to collagen (Figure S7) in the development of these microtissues, we introduced them to molds with posts that directed contraction along a primary axis (Figure 5A). The LC group exhibited contraction over 3 days to 56% the original thickness, whereas the HC group retained the shape of the mold (Figure 5B,C). With contraction of the LC group, increased cell density was observed at the periphery of contracted microtissues (Figure 5D,E). To compare the alignment induced by this contraction, we analyzed cell and nuclear morphology in the peripheral 5% of the construct. Here, actin and nuclear alignment, as well as nuclear aspect ratio increased in the LC group compared to the HC group (Figure 5F). When comparing distribution of alignment angles, the LC group had increased actin and nuclear alignment along the mold orientation compared to the HC group (Figure 5G). Taken together, these data show that the CCA (like collagen) can form a microtissue that exhibits alignment based on defined boundary conditions, establishing its utility in studies exploring cellular responses to ECM biochemical and biophysical cues during contractility-mediated tissue formation.

**FIGURE 6 |:**
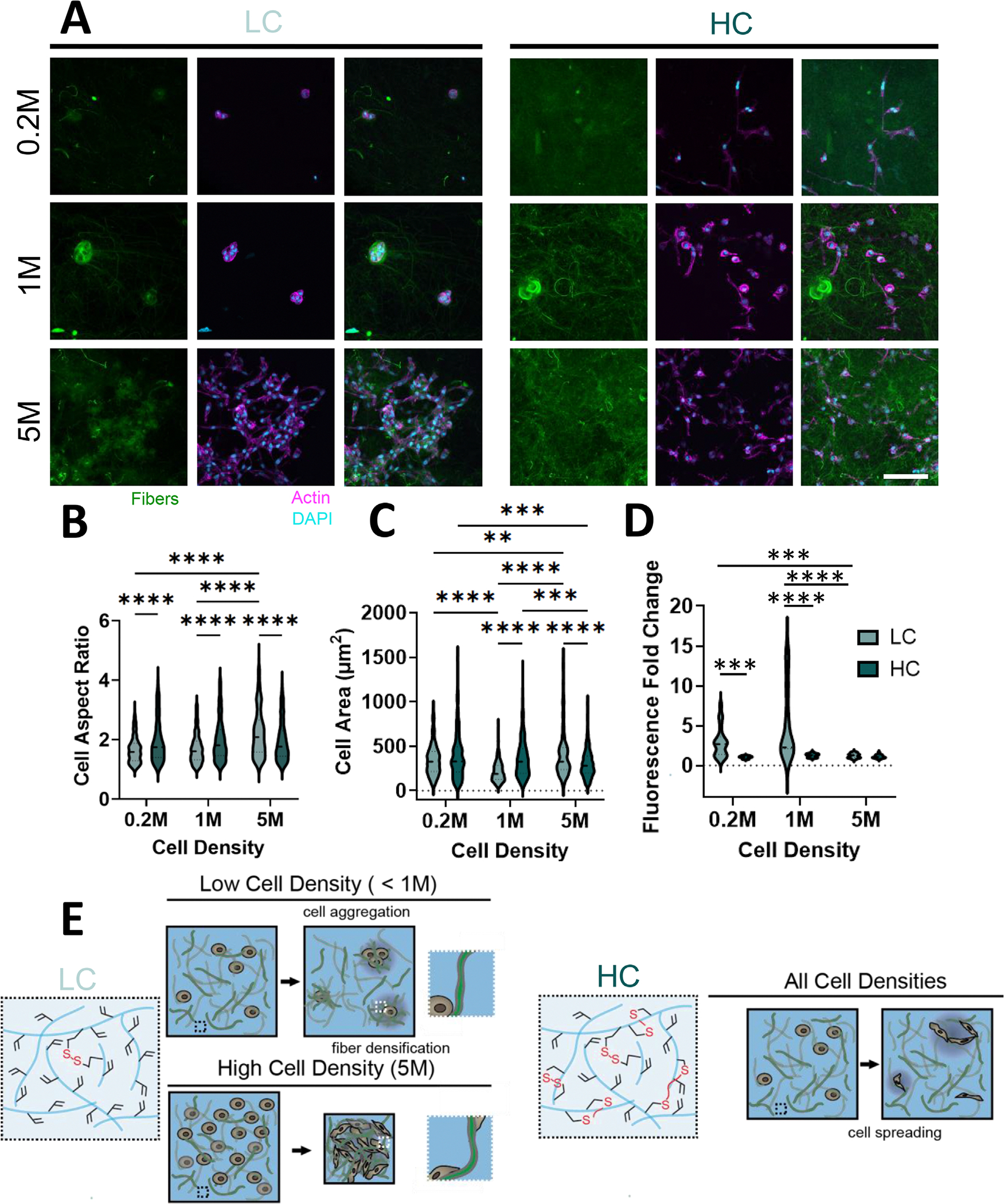
Microtissue contractility is supported on a cellular level through remodeling of pericellular microdomains. (A) Representative images of cell-ECM interactions in LC and HC groups with varying cell density (0.7% (w/v) CP; 5% (v/v) FF; LC: 5%, HC: 50% extent of CP crosslinking; 0.2M (million), 1M, or 5M cells/mL) at day 3. Scale bar = 100 μm. (B,C) Quantification of cell aspect ratio (B) and cell area (C). n≥ 100 cells per group. **p < 0.01, ***p < 0.001, ****p < 0.0001. Two-way ANOVA with Tukey post-hoc test. (D) Quantification of the fold change in fluorescent intensity of fibers colocalized with cells versus a random region without cells. *n*= 8–24 ROIs per group. ***p < 0.001, ****p < 0.0001. Two-way ANOVA on log-transformed data with Tukey post-hoc test. (E) Summary schematic of cell remodeling permissivity of LC and HC groups. Data are mean ± s.d.

To better understand how cells interact within the CCAs, we seeded cells at varying densities (0.2 million cells/mL (0.2M), 1 million cells/mL (1M), and 5 million cells/mL (5M)) to generate constructs with cell densities that did and did not contract. At low cell densities of 0.2M and 1M, cells in the LC group exhibited clustering (Figure 6A) but did not contract macroscopically (Figure 3A). This is reflected by the decreased cell area and aspect ratio, as well as nuclear aspect ratio in the LC compared to the HC groups (Figure 6B,C; Figure S8A). Clearly, at these cell densities, cells were unable to elongate to exert tractions to support contraction. The clusters in the LC group were also round, suggesting that cells may have migrated or contracted their pericellular environments to form localized cellular clusters. Interestingly, fiber fluorescence increased near the cell clusters in the low cell densities (at or less than 1M), which may suggest that cells are adhering and pulling on the fibers, leading to localized compaction (Figure 6D). Localized compaction without macroscopic contraction suggests that the cells may be remodeling their local environment, but are unable to exert the long-range force transmission necessary for macroscopic contraction given that the fibers themselves were not linked to each other or the CP. While the HC hydrogel is conducive to elongated cell morphologies, the covalently crosslinked CP may inhibit macroscopic tissue remodeling.

At the higher 5M cell densities, LC constructs contracted (Figure 3A) with cell area and aspect ratios that were higher than in the HC group (Figure 6A–C), suggesting increased cellular force transmission throughout the construct leading to macroscopic compaction. Additionally, at this higher cell density, cell clustering was not observed and local fluorescence was not higher than in areas without cells (Figure 6A,D). Improved force transmission may be due to increased cell density leading to stabilization of fibers at distal sites by other adherent cells, reducing the likelihood of a single cell (or group of neighboring cells) freely manipulating individual fibers and drawing them inwards toward the cell body. Thus, cells may themselves serve as effective crosslinks [36] in the material, enabling increased long range force propagation across multiple fiber domains to support macroscopic tissue contraction (Figure 5E,F). This would explain how the morphologies were rounder with lower cell densities in the LC groups but became elongated within high cell density constructs.

Of further note, the FFs in the CCA do not adhere to one another or to the surrounding CP. When cells were seeded at a density lower than the contraction threshold but given increased fibers to interact with, they did not contract (Figure S9). In this low cell density system, while cells may interact with fibers, these fibers are not stabilized elsewhere, allowing the cells to adhere and remodel the fibers without providing a substrate that supports cell-mediated elongation, traction, and construct contraction. Importantly, the cell-construct densification observed in the LC group at 5M was also observed in stiffness-matched collagen hydrogels regardless of cell concentration (Figure S10). This is likely because, unlike in the CCA constructs, collagen fibers are coupled to and entangled with one another and do not rely as heavily on cells to serve as coupling agents to enable macroscale construct compaction. In total, these studies suggest that, in the LC group, cells can adhere and remodel the fibers to a greater extent than in the HC group (Figure 6E). With this remodeling, cells in lower cell density constructs may exhibit localized aggregation and fiber densification. At higher cell densities, cells can stabilize fibers along several points of the fiber, enabling long-range force propagation that supports the observed macroscopic contraction.

## 3 | Conclusion

Here, we describe an engineered composite fibrous hydrogel in which cell-mediated contraction can be programmed based on continuous phase crosslinking to enable or resist macroscopic contraction through cell-adhesion to co-embedded adhesive fiber fragments. Previous studies have explored the use of fiber-hydrogel composites for increased mechanical properties [37–42] or mimicry of fibrous cues [43, 44]; however, these systems were not capable of contraction. In the LC CCA, we found that contraction required high cell densities and that cells may serve as crosslinkers between fiber fragments to first stabilize the construct and then allow long-range force propagation for macroscopic contraction. In contrast, the lower cell density constructs enabled cells to freely pull fibers, permitting localized microscopic compaction that did not translate to the macroscopic level. In the HC group, cells may find these fibers but be unable to dislodge them due to the CP properties. Notably, the only adhesive ligands present in the composite were on the FF and contraction was abrogated when integrin-binding was inhibited, suggesting that cells interact with and pull on fibers to mediate contraction. Future work will explore the timing of these events as well as apply this system toward understanding the role of cellular contraction against nascent fiber fragments that are present in the pericellular space of musculoskeletal tissues such as the meniscus as it transits from cell-rich disorganized to matrix-rich organized tissues. With slight modifications, this material may also be extended to bioprinting applications to achieve increasingly complex in-vitro structures through spatial or temporal tuning of CP crosslinking and thereby contraction. Ultimately, this material system will enable exploration of key inputs that guide tissue formation, providing new insights into regenerative strategies that may be leveraged toward tissue repair.

## 4 | Methods

All reagents were obtained from Sigma-Aldrich and Fisher Scientific unless otherwise specified.

### 4.1 | Macromer Synthesis

Macromers were prepared as described previously [45]. Briefly, HA was modified with either methacrylates (MeHA) or acrylates (AHA) [13] through reaction of HA with methacrylic anhydride or acrylic anhydride, respectively, at a pH 9–10 for 3 h at room temperature in deionized (DI) water. All polymers were purified via dialysis, lyophilized, and modification was confirmed using ^1^H NMR (Bruker Neo 400). MeHA and AHA of 34% and 98% modification, respectively, were used for all experiments (Figure S11).

To introduce adhesive moieties onto fibers, MeHA was modified with RGD (GCGYG*RGD*SPG, Genscript) through a Michael addition reaction between thiols (cysteine amino acids) and methacrylate groups [46]. Briefly, MeHA was dissolved at 2% (w/v) in 0.2 M (pH ~8) triethanolamine buffer (TEOA) containing 0.7 mM RGD peptide. The reaction was allowed to proceed overnight at 37°C and then purified via dialysis and lyophilized.

### 4.2 | Fabrication of Fragmented Fibers

Multifiber electrospinning was conducted as described previously [19]. The MeHA solution consisted of 3% (w/v) MeHA (RGD-modified) polymer, 2% (w/v) 900 kDa polyethylene oxide (PEO), and 0.05% (w/v) Irgacure 2959, all dissolved in DI water. Fluorescent fibers also had 4 mg/mL of fluorescent dextran (2 MDa) included within the precursor solution. The sacrificial PEO solution was similarly dissolved in DI water at a final concentration of 4% (w/v). The collecting mandrel (−5 kV) was placed 19 cm away from the source polymers, which were ejected at defined flow rates (PEO solution: 1.05 mL/h, 15 kV; MeHA solution: 0.5 mL/h, 18 kV) to produce a fiber mat. To stabilize these networks, fiber mats were exposed to ultraviolet light (15 mW cm^−2^, 320–390 nm, Omnicure S1500 UV, Spot Cure Systems) for 15 min, flipped, and exposed for another 15 min. To isolate individual fibers, the fiber mats were cut into ~2 mm^2^ sections and rehydrated in 6 mL of PBS in a scintillation vial. This solution was then repeatedly extruded and re-aspirated 40 times through an 18G needle (or 40 times sequentially through an 18G, 21G, and 23G needle for short FF studies). The resulting fragmented fiber solution was filtered sequentially through a 40 μm cell strainer, followed by a 5 μm (Pluriselect) cell strainer to isolate individual fragments. Fibers were rinsed 3x in PBS and pelleted at 10000×g for 3 min. Fiber stock concentrations were then determined by pelleting a portion of the solution at 10000×g in a packed cell volume (PCV) tube, in which the pelleted fiber fraction was considered to have a volume fraction of 100%, and the stock concentration of fiber solution was calculated accordingly. Fibers were imaged on an epi-fluorescent microscope (Leica DMI6000B). Fiber lengths were quantified (ImageJ) from >15 fields of view (671 × 567 μm) by manual tracing of fluorescent fibers in FIJI (Figure S1). Fiber diameters were quantified (ImageJ) as previously through quantifying full-width half maximum of a fluorescence intensity profile orthogonal to fiber length [46].

### 4.3 | Cell Isolation and Culture

Mesenchymal stromal cells (MSCs) were isolated from juvenile bovine joints (Research 87) as described previously [47]. Briefly, bone marrow was removed from femurs and tibias and centrifuged with DMEM with 2% penicillin/streptomycin/fungizone (PSF) and 2 g/L heparin. After centrifugation, samples were plated in DMEM supplemented with 10% Fetal Bovine Serum (FBS) and 1% PSF (penicillin, streptomycin, and amphotericin) until 80% confluency of initial colonies and then stored in liquid nitrogen (90% FBS, 10% dimethylsulfoxide). For in vitro studies, MSCs were used between passages 1–4 and used on passage 1 for RNAseq studies.

### 4.4 | Composite Contractile Assembly Fabrication

Fibrous composites were assembled by mixing constituents (the continuous phase (CP) of AHA macromers and the fragmented fibers (FF)) and cells at designated concentrations in a solution of Media 199 (pH 8.5) supplemented with 10% FBS, 1% PSF, 2.5 mM tris(2-carboxyethyl)phosphine (TCEP) and 25 mM 4-(2-hydroxyethyl)-1-piperazineethanesulfonic acid (HEPES) [48, 49]. For fluorescent constructs, fluorescent fibers were added to unlabeled fibers at a ratio of 1:100. Dithiothrietol (DTT) was added as a dithiol crosslinker and the solution was allowed to undergo a Michael Addition reaction in pH of 8.5 for 1 h at 37°C, 5% CO_2_. The added DTT concentration was calculated with an assumption that each thiol would react with and consume an available acrylate group. As such, we assume that 100% consumption would be achieved through addition of equivalent mM thiol groups to mM acrylates (based on initial polymer modification). Lower thiol concentrations were added proportionally to achieve lower consumptions. After crosslinking, hydrogels were cultured in basal media (high glucose DMEM, supplemented with 10% FBS and 1% PSF) at 37°C and 5% CO_2_.

### 4.5 | Collagen Gel Fabrication

Collagen hydrogels were fabricated as previously described [19]. For fluorescent collagen, non-labeled type 1 Bovine telocollagen (Advanced Biomatrix) was mixed with FITC-labeled type 1 bovine collagen to create a stock solution of 12% (v/v) fluorescent and 88% (v/v) non-fluorescent collagen. This stock solution or non-labeled telocollagen were added with cells, basal media and associated neutralization solution (Advanced Biomatrix) or with NaOH to achieve the desired final collagen concentration (2.5 mg/mL unless otherwise specified) at a pH of ~7. Collagen hydrogels underwent gelation for 1 h at 37°C, 5% CO_2._ Afterwards, hydrogels were cultured in basal media.

### 4.6 | In Vitro Contraction Assays

Constructs were created by embedding MSCs in precursor solutions and pipetting into circular polydimethylsulfoxide (PDMS) molds with a diameter of 2 mm and thickness of 1 mm. Unless otherwise specified, hydrogels were formed at 0.7% (w/v) CP, 5% (v/v) FF, 5% acrylate consumption (for LC group) or 50% consumption (for HC group), and 5 × 10^6^ cells/mL. For studies in which cell contractility was modified, 10 μm blebbistatin was identified as a concentration that abrogated traction in collagen gels (Figure S3) but maintained cell viability. This concentration vs. DMSO at 0.1% (v/v) for vehicle control were compared for traction studies. For soluble RGD, 5 μm soluble RGD was identified as a concentration that reduced integrin adhesion while maintaining cell viability. DMSO at 2% (v/v) was used as vehicle control for soluble RGD studies. At specified time points, hydrogels were imaged with a bright field microscope (Nikon Eclipse TS100) and construct area was quantified in ImageJ and normalized to area on Day 0. Live/Dead imaging was performed by treating constructs with calcein AM (2 μM) and ethidium homodimer-1 (4 μM) for 30 min at 37°C and imaging on an upright (Leica TCS SP5) or inverted confocal microscope (Zeiss Axio Observer 7).

### 4.7 | Mechanical Characterization (Rheology)

Hydrogels were formed as described and deposited on the bottom plate of a rheometer immediately after mixing. A Discovery HR20 (TA Instruments) rheometer was fitted with a 20 mm diameter cone geometry and placed at a 20 μm gap. Hydrogels were allowed to crosslink while applying an oscillating torque at 1 Hz and 1% strain for 60 min at 37°C unless otherwise specified. After crosslinking, strain-stiffening was assessed through strain sweeps (1 Hz, 0.001%–1000% strain).

### 4.8 | Construct Stability

CCA and collagen hydrogels were formed with 5.78 mg/mL TRITC-dextran (500,000 Da MW), hydrated and stored at 37°C, 5% CO_2._ Within each individual experiment, hydrogels were imaged daily up to 3 days with consistent exposure settings across all samples using a Zeiss AxioZoom V16 fluorescence microscope at 20× magnification.

### 4.9 | Alignment Studies

Constructs were seeded in custom 3D PDMS molds (Figure S12) with 2 boundary posts, which were created from positive molds and 3D printed with biomed clear resin on a Form 3 printer (FormLabs). Constructs were allowed to contract for 3 days, fixed for 30 min with 10% neutral buffered formalin and embedded in 5% (w/v) low-melting agarose. Constructs were then permeabilized with 0.1% (v/v) Triton X for 30 min and actin was stained with phalloidin 647 in 1% (w/v) BSA for 2 h at room temperature or at 4°C overnight. Prior to imaging, constructs were stained with 20 μm Hoechst 33342 for 30 min at room temperature. Z-stack images were acquired on an upright confocal microscope. Actin and nuclear alignment frequency distributions of max projections were obtained using the OrientationJ FIJI plugin (Gaussian window). Alignment indices were calculated as the normalized fraction found within twenty degrees of the axis of tension compared to the expected fraction without any alignment [50]. Indices from 2 ROIs were averaged per construct. Nuclear area and aspect ratio were assessed in FIJI (Moments threshold, analyzed particles were greater than 50 μm to remove artifacts and less than 200 μm to filter overlapping nuclei that were incorrectly identified).

### 4.10 | RNA Isolation, Sequencing, and Analysis

Four to six constructs (comprised of tissue excised between the boundary posts) per experimental group per donor were isolated and combined. RNA extraction was performed according to the manufacturer’s protocol (Maxwell RSC simplyRNA blood kit, Promega). RNA concentration was quantified (Nanodrop) and analyzed (Tapestation, Agilent). RNA libraries were prepared from 100 ng of RNA (RIN>8, Illumina Stranded mRNA RNAseq kit) and sequenced (NovaSeq 6000 sequencer). Quality control was performed (Tapestation and Qubit). Read mapping to the cow genome (bosTau9) was conducted using STAR on the Galaxy Europe platform, followed by differential gene expression analysis with DESeq2. Genes were classified as differentially expressed if the fold change between experimental groups exceeded 2 and the Benjamini-Hochberg adjusted p-value (p-adj) was below 0.05. Transcripts per million (TPM) values were derived from the mean normalized fragment counts generated by DESeq2 across all samples. Gene ontology and pathway analyses were carried out using the functional annotation tools Enrichr [51] and g:Profiler [52].

### 4.11 | Cell-ECM Interaction Studies

Cells were seeded at a density of 0.2 × 10^6^, 1 × 10^6^, and 5 × 10^6^ MSCs/mL into circular PDMS molds (2 mm thickness, 3 mm diameter). At acrylate consumptions at or less than 5%, 5% (w/v) low melting agarose was added on top of the constructs after fixation but prior to staining to stabilize the constructs for further analysis. Constructs were stained for actin and nuclei and imaged as above. Quantification of nuclear and cell morphology was performed using CellProfiler version 4.2.8. Nuclei were visualized using DAPI staining and segmented using the Otsu thresholding algorithm. The cell body was identified based on Alexa Fluor 647-labeled actin using a similar Otsu-based segmentation approach. To define the cell boundary, actin signals were propagated outward from the identified nuclei using the Propagation module. Cells and nuclei touching the image boundaries were excluded from analysis. Cells with an area below 50 μm^2^ or above 1500 μm^2^ were excluded. Nuclei with an area below 20 μm^2^ or above 200 μm^2^ were excluded. For each identified object, the major and minor axes were measured, and the aspect ratio (long to short axis) was calculated to represent nuclear/actin elongation. Fluorescent fold change was quantified as the normalization of fiber fluorescent intensity colocalized to each cell (actin) by the average fluorescence intensity in a 50 × 50 μm region without cells in the same image.

### 4.12 | Statistical Analysis and Data Presentation

All statistical analyses were performed using Microsoft Excel and GraphPad 20. The robust regression and outlier removal method, which eliminates outliers based on a fitted curve that is not originally influenced by data, was used to remove outliers prior to performing statistical tests. Data or log-transformed data were tested for normality with the Shapiro-Wilk test. When using a Student’s *t*-test or ANOVA, an *F*-test or Brown-Forsythe test was performed, respectively, to ensure homogeneity of variance. For statistical comparison between two experimental groups, a two-tailed unpaired Student’s *t*-test for parametric data (or Mann-Whitney test for nonparametric data) was used. For one-factor comparisons between more than two groups, a one-way ANOVA with Tukey post hoc testing for parametric data (or Kruskall-Wallis with Dunn’s multiple comparisons for nonparametric data) was used. For one-factor studies in which samples satisfied normality but did not satisfy homogeneity of variance, a Welch’s *T*-test or a Welch’s ANOVA with Dunnett’s T3 multiple comparison test was used for two or more than two groups, respectively. All two-factor analyses were conducted with two-way ANOVAs with Tukey post-hoc testing regardless of normality and homogeneity of variance, as this test provided the most biologically relevant analyses. A *p*-value<0.05 was considered significant. When no experimental groups were significant, ns was used to denote no statistical significance. For the ANOVA analysis, a straight line between significant groups was denoted and unlabeled groups have no statistical significance. For the circular alignment data, comparisons were assessed with the Watson-Wheeler test. Sample n values were included within figure captions and all data were reported as the mean ± standard deviation. Schematics were designed with Adobe Illustrator.

## Supplementary Material

Supplemental info

Additional supporting information can be found online in the Supporting Information section.

**SupportingFile**: adfm74338-sup-0001-SuppMat.pdf.

## Data Availability

The data that support the findings of this study are available from the corresponding author upon reasonable request.
