# Supplementary material for: Controlling 3D Contractility via Engineered Fibrous Hydrogel Composites
Source: Adv Funct Mater. Author manuscript; Available in PMC 2026 Jun 3. (PMC13229568; doi:10.1002/adfm.202524101)
Supplement: Supplemental info [file NIHMS2175060-supplement-Supplemental_info.docx]

**Supplemental Information:**

**Controlling 3D contractility via engineered fibrous hydrogel composites**

Karen L. Xu^1,2,3^, Yuqi Zhang^1,2,3^, Alysse DeFoe^4,5^, Georgios Kotsaris^1,2,3^, Brendan Stoeckl^1,3^, Matthew D. Davidson^2,4,5^, Jason A. Burdick^1,2,4,5^, Robert L. Mauck^1,2,3^

^1^Department of Bioengineering, University of Pennsylvania, Philadelphia, PA, USA

^2^Center for Engineering Mechanobiology, University of Pennsylvania, Philadelphia, PA 19104, USA

^3^McKay Orthopaedic Research Laboratory, Department of Orthopaedic Surgery, Perelman School of Medicine, University of Pennsylvania, Philadelphia, PA, USA

^4^BioFrontiers Institute, University of Colorado Boulder, Boulder, CO, USA

^5^Department of Chemical and Biological Engineering, University of Colorado Boulder, Boulder, CO, USA

Corresponding Authors: [lemauck@pennmedicine.upenn.edu](mailto:lemauck@pennmedicine.upenn.edu) and jason.burdick@colorado.edu

**Supplemental Information**

**Supplemental Figure 1:** **Characterization of length and thickness of Fragmented Fibers (FF)** (**A**) Representative image of fluorescent fiber image used for quantification (Scale bar = 100 $\mu$m). Quantification of fiber length (**B**, summary plot; **C**, histogram of fiber length distribution). n= 138 FF. (**D,E**) Representative image (**D**, Scale bar = 50 $\mu$m) of fluorescent fiber with yellow line denoting distance in corresponding fluorescent intensity profile (**E**). (**F**) Quantification of fiber thickness. n= 106 FF. Data are mean ± s.d.

**Supplemental Figure 2: FF are necessary for CCA contraction. (A)** Representative images (**A,** Scale bar = 400 $\mu$m) and quantification (**B**) of CCA with increased AHA at day 3 (1% (w/v) CP; 0 or 5 (v/v)% FF; 5% acrylate consumption; 5 x10^6^ cells/mL, n= 7-8 constructs). ****p<0.0001. Two-tailed unpaired Student’s t-test. Data are mean ± s.d.

**Supplemental Figure 3**: **Blebbistatin abrogates collagen contraction.** (**A-C**) Representative contraction images (**A**, Scale bar = 400 $\mu$m), cell viability images (**B**, green: live cells, red: dead cells, Scale bar = 100 $\mu$m), and quantification (**C**) of contraction from panel A of collagen gels with vehicle or blebbistatin. n= 8-10 samples per group. ****p<0.0001. Mann-Whitney test. Data are mean ± s.d.

Inflammatory Response

ECM Organization

**B**

**D**

**E**

**C**

Integrin-Mediated

Signaling Pathway

ECM Organization

Actin-Based Cell Projection

**A**

**Supplemental Figure 4: Transcriptomic analysis suggests key differences in cell states in CCA vs. collagen.** (**A**) Volcano plot highlighting differentially expressed genes between CCA vs. collagen control groups (red: upregulated; blue: downregulated). (**B,C**) Bar graphs (**B**) showing gene ontology groups downregulated between CCA and collagen groups (BP: biological process, CC: cellular component, and MF: molecular function) and corresponding heat maps (**C**) for integrin-mediated signaling pathway (left), ECM organization (middle) and actin-based cell projection (right) with hierarchical clustering of treatment groups by donor (column) and genes (rows). (**D,E**) Bar graphs (**D**) showing gene ontology groups upregulated between CCA and collagen groups and corresponding heat maps (**E**) for ECM organization (left) and inflammatory response (right).

**Supplemental Figure 5**: **Varying crosslinking extent of CP does not reduce cell viability.** Representative images of cell viability (green: live cells, red: dead cells) from >12 ROIs across 6 constructs per group (Scale bar = 100 $\mu$m).

**Supplemental Figure 6: Constructs remain intact after 3 days.** Representative images of acellular hydrogels at day 3 with embedded TRITC-dextran (top row: intensity-matched images; bottom-row: images with increased post-process gain). Scale bar = 400 $\mu$m.

**Supplemental Figure 7**: **Alignment of collagen microtissues is based on contraction along an axis of tension.** (**A**) Representative images of collagen contraction (dashed white line represents ROIs for analysis in panel B-E). Scale bar = 100 $\mu$m. (**B-E**) Quantification of actin alignment (**B**, n= 6 constructs), nuclear alignment (**C**, n= 6 constructs), nuclear area (**D**, n= 31 cells), and nuclear aspect ratio (**E**, n= 31 cells). (**F**) Frequency distribution of orientation of actin (left panel) and nuclei (right panel). n= 12 ROIs across 6 constructs per experimental group. Data are mean ± s.d.


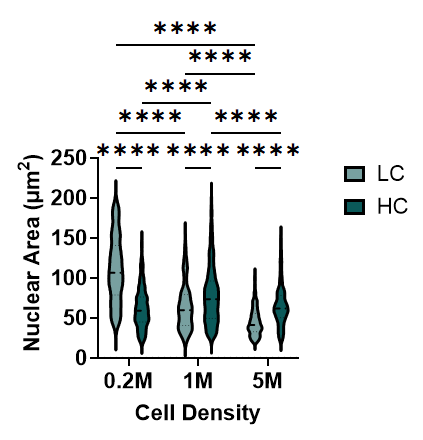


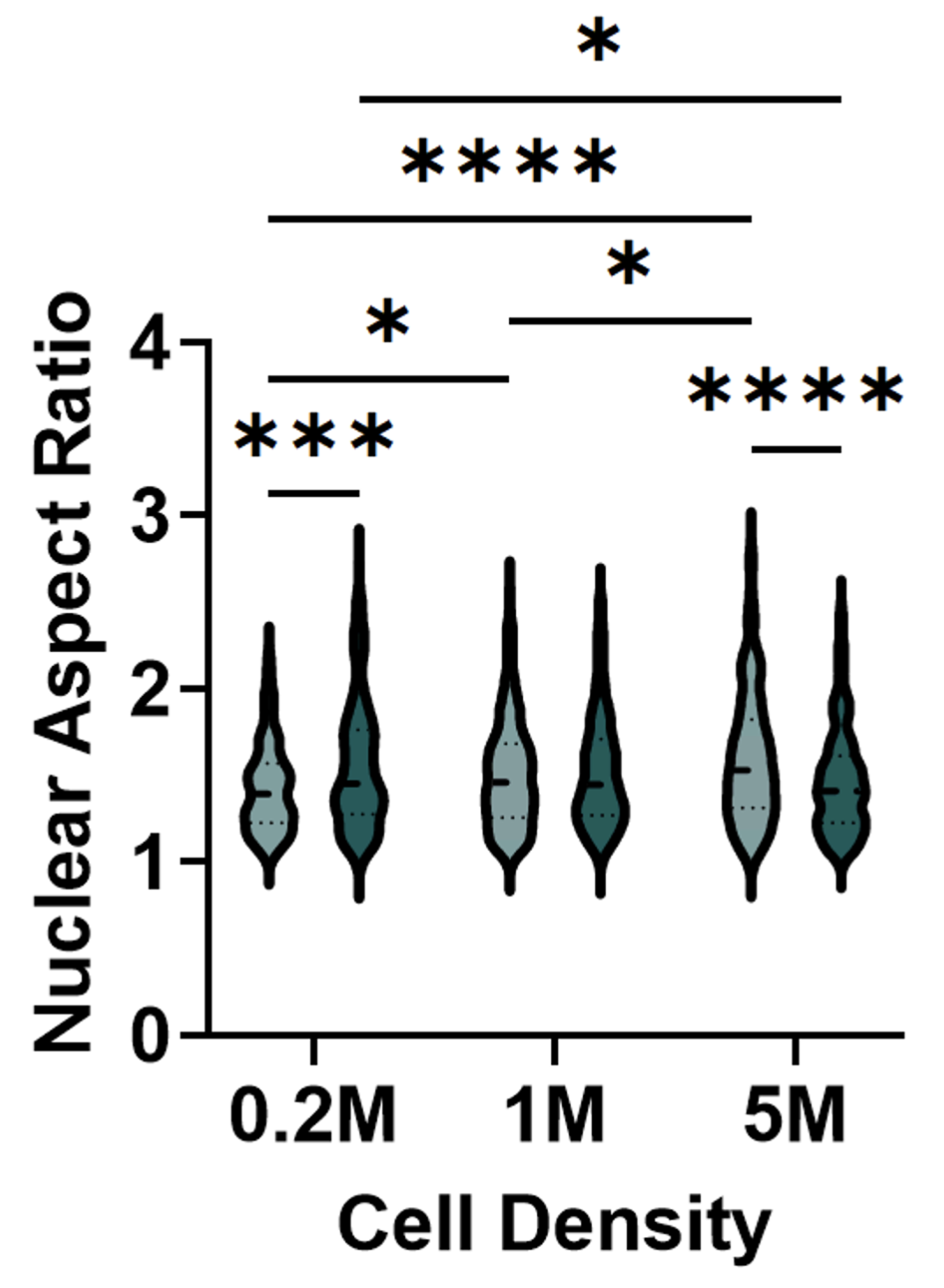


**Supplemental Figure 8**: **Nuclear morphologies in response to cell density and CCA group.** Quantification of nuclear aspect ratio (left panel) and nuclear area (right panel). n$\geq$ 100 nuclei per group. *p<0.05, ***p<0.001, ****p<0.0001. Two-way ANOVA with Tukey post-hoc test. Data are mean ± s.d.

**Supplemental Figure 9**: **Increasing fiber populations in low cell-density constructs does not enable contraction.** (**A-B**) Representative images (**A**, Scale bar = 400 $\mu$m) and quantification (**B**) of CCA (0.7% (w/v) CP; 5%, 10%, 20% (v/v) FF; 5% acrylate consumption; 1 x10^6^ MSCs/mL, n= 8-24 constructs) over time. ns indicates no statistical significance. Kruskall-Wallis with Dunn’s multiple comparisons. Data are mean ± s.d.

**Supplemental Figure 10**: **Stiffness-matched collagen groups exhibit high construct densification.** (**A,B**) Representative rheological time sweep (**A**, storage (G’) and loss (G”) modulus, 1 Hz, 1% strain, left panel) and final rheologic mechanical properties of assemblies (**B**, LC: 5% extent of CP Crosslinking). n= 3 samples per group. ns indicates no statistical significance. Two-tailed unpaired Student’s t-test between G’. (**C**) Representative max projections of cells at specified densities in 0.16 mg/mL collagen constructs. Scale bar = 50 $\mu$m. n= 2 samples per group. Data are mean ± s.d.

**Supplemental Figure 11: ^1^H NMR spectra of synthesized MeHA and AHA. (A)** Chemical structure and ^1^H NMR spectrum of methacrylated hyaluronic acid (MeHA). Modification of MeHA was determined by the integration of the vinyl group of the methacrylate (2H, δ: 5.6-5.7 ppm, 6.0-6.2 ppm, red) normalized to the hyaluronic acid backbone (10H, 3.1-4.1 ppm, gray). (**B**) Chemical structure and ^1^H NMR spectrum of acrylated hyaluronic acid (AHA). Modification of AHA was determined by the integration of the vinyl group of the acrylate (3H, δ: 5.9-6.1 ppm, 6.1-6.3 ppm, 6.3-6.5 ppm, blue) normalized to the hyaluronic acid backbone (10H, 3.1-4.1 ppm, gray).

**Supplemental Figure 12: Positive 3D-printed molds.** Negative PDMS molds were constructed from 3D-printed positive molds (**A**). All dimensions (**B**) for the positive molds are in millimeters.
